# Screening and Treatment for Alcohol, Tobacco and Opioid Use Disorders: A Survey of Family Physicians across Ontario

**DOI:** 10.1371/journal.pone.0124402

**Published:** 2015-04-29

**Authors:** Genane Loheswaran, Sophie Soklaridis, Peter Selby, Bernard Le Foll

**Affiliations:** 1 Translational Addiction Research Laboratory, Campbell Family Mental Health Research Institute, Centre for Addiction and Mental Health, Toronto, Ontario, Canada; 2 Department of Pharmacology, University of Toronto, Toronto, Ontario, Canada; 3 CAMH Education, Centre for Addiction and Mental Health, Toronto, Ontario, Canada; 4 Department of Psychiatry, University of Toronto, Toronto, Ontario, Canada; 5 Department of Family and Community Medicine, University of Toronto, Toronto, Ontario, Canada; 6 Ambulatory Care and Structured Treatments, Centre for Addiction and Mental Health, Toronto, Ontario, Canada; 7 Dalla Lana School of Public Health, University of Toronto, Toronto, Ontario, Canada; VU University Medical Center, NETHERLANDS

## Abstract

**Introduction:**

As a primary point of contact within the health care system, family physicians are able to play a vital role in identifying individuals with substance use disorders and connecting them to the appropriate treatment. However, there is very little data available on whether family physicians are actively screening for and treating substance use disorders. The objective of the current survey was to assess whether family physicians in Ontario are screening for alcohol, opioid and tobacco use disorders, using validated tools and providing treatment.

**Methods:**

An online survey consisting of a series of 38 primarily close-ended questions was circulated to family physicians in Ontario. Rates of screening for alcohol, opioid and tobacco dependence, use of validated tools for screening, providing treatment for dependent individuals and the current barriers to the prescription of pharmacotherapies for these drug dependences were assessed.

**Results:**

The use of validated screening tools was limited for all three substances. Screening by family physicians for the substance use disorders among adolescents was much lower than screening among adults. Pharmacotherapy was more commonly used as an intervention for tobacco dependence than for alcohol and opioid dependence. This was explained by the lack of knowledge among family physicians on the pharmacotherapies for alcohol and opioid dependence.

**Conclusions:**

Findings from the current study suggest there is a need for family physicians to integrate screening for substance use disorders using validated tools into their standard medical practice. Furthermore, there is a need for increased knowledge on pharmacotherapies for alcohol and opioid use disorders. It is important to note that the low response rate is a major limitation to this study. One possible reason for this low response rate may be a lack of interest and awareness among family physicians on the importance of screening and treatment of substance use disorders in Ontario.

## Introduction

The harmful use of psychoactive substances is a well-known public health problem in Canada and world-wide [1–5]. Recent findings from US studies revealed that the lifetime prevalence of alcohol and illicit drug abuse in US adults is approximately 13–18% and 8% respectively [6]. In 2002, substance–related problems were related to 47,000 deaths, the loss of 768,000 life-years and 4.15 million hospital days in Canada [2, 7].

Substance use disorders can attract a negative social response and stigmatization by society and even family physicians [8–10]. In addition, the use of many of these substances is illegal. Therefore, many substance-related problems are often under-reported to healthcare providers. Since only a small proportion of individuals who have substance use disorders actively seek treatment, screening among healthcare providers, and notably by family physicians, is important to detect those disorders. Additionally, screening for possible illnesses and disorders and ensuring that patients receive the appropriate care is central to the role of family physicians. They act as a vital link to connect individuals under their care to the appropriate interventions. As many individuals with these disorders may not seek help on their own, the ability of family physicians to identify and treat these disorders is crucial.

There is limited existing information on the current screening and treatment practices of family physicians for substance use disorders in Canada. One study using an anonymous mail survey revealed that there was a lack of regular screening for substance use disorder during general medical examinations [11]. In a study using two focus-group interviews of 12 family physicians practicing in London, Ontario, physicians reported that a lack of systematic strategy and actual materials were barriers to identifying and managing patients who use alcohol [12]. Findings from other Canadian surveys in the past also revealed that screening and treating of alcohol abuse and dependence among family physicians was lacking [13, 14]. However these studies did not ask about screening and treatment for the other substance use disorders. To investigate this issue, we conducted an online survey covering alcohol, tobacco and opioid use disorder screening and treatment practices among family physicians in Ontario. These drugs were selected based on alcohol and tobacco’s high rates of dependence and significant contributions to morbidity and mortality worldwide [3, 15, 16]. Additionally, opioids (including prescription and non-prescription) were included in the survey given the increase in opioid abuse, specifically prescription opioid abuse in recent years [17, 18]. Findings from the current study suggest that there is a need to increase awareness among family physicians on the importance of screening and treatment of substance use disorders in Ontario.

## Methods

Family physicians (n = 11,000) throughout Ontario were given the opportunity to participate in the online survey through a link that was published in the monthly News Brief of the Ontario College of Family Physicians in January, June and August in 2012. The News Brief is emailed to the members of the Ontario College of Family Physicians, consisting of approximately 11,000 individuals.

The anonymous online survey was developed using the Centre for Addiction and Mental Health’s *Checkbox* survey software (http://www.checkbox.com/). The survey consisted of a series of 38 questions (mainly closed-ended). The survey included questions on demographics, years of practice, type of practice (community health centre, family health team, private practice, hospital or other), number of alcohol, tobacco and opioid dependent individuals under care as well as questions about screening and treatment practices. The questionnaire was piloted among five physicians to ensure clarity. The survey was approved by the Research Ethics Board at the Centre for Addiction and Mental Health on September 7, 2011 (REB Protocol #021/2011). Given that this was an electronic survey, participants were asked to provide their informed consent through a checkbox feature prior to commencing the online survey.

## Results

The survey was completed by 119 family physicians throughout Ontario. The demographic information of the physicians is provided in Table 1.

**Table 1:** Demographics of Family Physicians Surveyed.

Survey Strata	Number (%) of Family Physicians
**Gender**	
Male	84 (71)
Female	35 (29)
**Years of Practice as Family Physician**	
<5 years	44 (37)
5–10 years	13 (11)
11–20 years	25 (21)
21–30 years	23 (19)
>30 years	14 (12)
**Work Organization**	
Community Health Centre	11 (9)
Family Health Team	36 (30)
Private Practice	34 (29)
Hospital	11 (9)
Other	27 (23)
**Number of Days per Week of Practice**	
1 day	5 (4)
2 days	5 (4)
3 days	16 (14)
4 days	33 (28)
5 days	54 (45)
6 days	5 (4)
7 days	1 (1)
**Substance dependent patient(s) under care**	
Tobacco	116 (97)
Alcohol	105 (88)
Opioid	99 (83)

Most physicians reported that they do some form of screening among adults: 96% of physicians for tobacco and alcohol use disorders and 79% for opioid use disorders. However, the use of validated tools for screening was low. Only 41% of family physicians reported that they use validated tools with some patients to screen for alcohol use disorders/dependence, and only 15% and 25% said they use validated tools with some patients to screen for tobacco and opioid dependence respectively. Only 7% of family physicians reported that they use validated tools with all patients to screen for tobacco and alcohol use disorders. Physician screening for all three of the drug use disorders was lower for adolescents (<18 years of age) in comparison to adults. Screening was particularly low for opioid use disorders among adolescents, with only 44% of physicians doing any form of screening for adolescents for opioid use disorders in comparison to 73% and 70% screening for tobacco and alcohol use disorders respectively.

The use of various interventions for individuals with tobacco, alcohol and opioid dependence is presented in *Table 2,* while *Table 3*summarizes physicians’ knowledge on the pharmacotherapies and Table 4 the barriers for prescription reported by physicians.

**Table 2:** Use of Interventions for Substance Dependences.

Intervention	Tobacco (% of Family Physicians)	Alcohol (% of Family Physicians)	Opioid (% of Family Physicians)
Provide Information About Risks	96	93	88
Brief Motivational Interviewing	91	89	74
Cognitive Behavioral Therapy	20	16	15
Pharmacotherapy	98	39	37

**Table 3:** Family Physicians’ Knowledge on the Pharmacotherapies for Substance Dependences.

Knowledge in Prescribing Pharmacotherapies	Tobacco Number (%) of Family Physicians	Alcohol Number (%) of Family Physicians	Opioid Number (%) of Family Physicians
Sufficient knowledge on *ALL* pharmacotherapies and comfortable prescribing	38 (32)	5 (4)	5 (4)
Sufficient knowledge on *SOME* pharmacotherapies and comfortable prescribing	77 (65)	35 (29)	33 (28)
Some knowledge on *ALL* of the pharmacotherapies but not comfortable prescribing	1 (1)	10 (8)	13 (11)
Some knowledge on *SOME* of the pharmacotherapies but not comfortable prescribing	3 (3)	51 (43)	57 (48)
No knowledge about pharmacotherapies and not comfortable prescribing	0 (0)	6 (5)	10 (8)
Did not know that Health Canada approved pharmacotherapies were available	0 (0)	12 (10)	1 (1)

**Table 4:** Barriers to Prescribing Pharmacotherapies for Substance Dependences.

Barriers	Tobacco Number (%) of Family Physicians	Alcohol Number (%) of Family Physicians	Opioid Number (%) of Family Physicians
Lack of knowledge about pharmacotherapies	28 (24)	105 (88)	97 (82)
Lack of reimbursement by government to patients for cost of pharmacotherapies	N/A	60 (50)	51 (43)
Lack of patient compliance	76 (64)	56 (47)	70 (59)
Lack of effectiveness of existing pharmacotherapies	40 (34)	29 (24)	27 (23)
Concerns about risks and side effects of existing pharmacotherapies	65 (55)	42 (35)	62 (52)
No barriers	9 (8)	0 (0)	2 (2)
Other barriers	11(9)	7 (6)	18 (21)

Of the respondents, 58% reported that an increase in knowledge through brief training would increase their use of pharmacotherapies for tobacco dependence while 93% reported this for alcohol dependence and 85% for opioid dependence. 70% and 55% of physicians reported that governmental reimbursement for the cost of pharmacotherapies would increase their use of pharmacotherapies as an intervention, for alcohol and opioid, respectively.

## Discussion

Findings from the survey revealed that while most physicians do some form of screening for the substance use disorders, the use of validated tools for screening is low. Screening of adolescents for the three substance use disorders was lower than for adults. Screening for opioid disorders among adolescents was particularly low. The use of pharmacotherapies was generally low, with the exception of tobacco dependence, for which the use of pharmacotherapies was very common. This was explained by the lack of knowledge among family physicians on the pharmacotherapies for alcohol and opioid dependence. In addition to lack of knowledge on these pharmacotherapies, lack of government reimbursement for the cost of pharmacotherapies for alcohol and opioid use disorders was commonly reported as a barrier to the prescription of pharmacotherapies by family physicians.

Despite the majority of physicians reporting that they screen for the three substance use disorders, the use of validated tools for screening was low. Screening through formal questionnaires is the best way to identify individuals who have substance use disorders. Health Canada suggests the use of screening instruments that are brief and that have good reliability and validity [19]. The CAGE (for alcohol use disorders)and CAGE-AID (a version of the CAGE questionnaire modified to include drug use) questionnaires are recommended by Health Canada as brief screening instruments [19] and belong to Health Canada’s *Level 1 Screening* procedures (the least time consuming and the most appropriate for use by family physicians). These questionnaires consist of only four questions each that can be quickly and easily administered by family physicians. Increasing awareness among family physicians on the value of using these brief validated tools to screen for the substance use disorders can increase the likelihood of correctly identifying individuals with substance use disorders and connecting them with the appropriate intervention.

The lack of screening for the substance use disorders among adolescents in comparison to adults is particularly concerning given the high prevalence of alcohol and illicit drug use in adolescence [20]. The use of substances early in life has been linked to an increased risk for substance dependence, medical and psychiatric disorders and mortality [21–24]. Screening of adolescents to identify problematic substance use is important to ensure they receive the appropriate interventions early on [25]. While substance abuse screening instruments targeted for the adolescent population are available [26–33], the majority are too long to be used as a brief screening tool by family physicians [34]. The CAGE-AID has demonstrated validity in identifying adolescents with substance use disorders in mental health care [35] but further research is required to identify the validity of this questionnaire in primary health care settings. There is an urgent need for validated brief screening tools for adolescents that are appropriate for use by family physicians. Also, training for family physicians should emphasize the importance of regular screening for substance use disorders among adolescents.

Tobacco use was the most commonly screened (using validated tools) and treated (using pharmacotherapies and behavioural therapies) drug use disorder by the family physicians in our study. Screening and treatment for alcohol and opioid use disorders was much lower in comparison. This discrepancy between the screening and treatment practices between tobacco and alcohol and opioid use disorders may be attributable to the numerous public health initiatives to increase awareness of the harms of tobacco use and facilitate smoking cessation [36–38]. In March 2010, the Ontario Tobacco Research Unit published a report entitled “*The Next Stage*: *Delivering Tobacco Prevention and Cessation Knowledge through Public Health Networks”*, which included information on clinical best practice to facilitate smoking cessation [39]. Programs targeted at improving physician competency to treat tobacco dependence have also been effective in improving treatment practices [40]. However, given the voluntary nature of these programs, only a limited number of physicians obtain this training. Given the depth and breadth of the numerous public health initiatives aimed at increasing awareness, there are still gaps in the expertise of family physicians on interventions for tobacco dependence. This is supported by our survey findings revealing that only 32% of the physicians surveyed reported that they have sufficient knowledge on all the pharmacotherapies and felt comfortable prescribing.

Along with behavioral interventions, pharmacotherapies have demonstrated both efficacy and safety for the treatment of substance use disorders [41, 42]. While pharmacotherapies may not be the ideal intervention for all individuals with substance use disorders, they are appropriate and effective intervention for a subset of these individuals. Therefore, it is important that family physicians are knowledgeable on the currently available pharmacotherapies for the alcohol, tobacco and opioid dependences. Lack of physician knowledge about pharmacotherapies for alcohol and opioid dependence was the most commonly reported barrier to the prescription of these pharmacotherapies. This is further supported by 93% and 85% of family physicians reporting that increases in their knowledge on pharmacotherapies through brief training would increase their use of pharmacotherapies for alcohol and opioid dependence respectively. Public health efforts to increase knowledge on the pharmacotherapies and other interventions to treat alcohol and opioid use disorders will serve as a valuable step to increasing access to treatment and decreasing the harms caused by alcohol and opioid use disorders. It is clear from findings from the current survey that there is a need for specialized training on the treatment of substance use disorders. While voluntary training programs increase the competency of those who seek this training, there is an urgent need for a more global training of family physicians in this area. Improving the knowledge provided on treating substance use disorders through the Canadian medical school curriculum and/or during residency training for family physicians may be the best way to address this need.

Lack of government reimbursement for the cost of pharmacotherapies for alcohol and opioid use disorders was another highly reported barrier to the prescription of pharmacotherapies by family physicians. While prescription pharmacotherapies for tobacco dependence are now paid under the Ontario Drug Benefit (ODB) plan, naltrexone and acamprosate for alcohol dependence are only covered under the “exceptional access” part of the ODB. Methadone is fully covered under the ODB while suboxone is only covered under “limited use”. In the current survey, 70% of physicians who were aware that pharmacotherapies for tobacco dependence are now covered by the ODB reported that they now prescribe these pharmacotherapies to tobacco dependent individuals more often because of this change. Providing full coverage for the pharmacotherapies for alcohol and opioid dependence under the ODB will eliminate this barrier to the prescription of pharmacotherapies and facilitate prescribing practices for these pharmacotherapies.

Of particular interest currently are family physicians’ screening and treatment practices for prescription and non-prescription opioids. In 2007, the Annual Report by the International and Narcotics Control Board cautioned that “abuse of prescription drugs [will] surpass illicit drug abuse globally” [43]. The abuse of prescription opiates is becoming an increasing problem in the United States and Canada [18, 44–53]. There has been an increase in reports of non-medical use of opioids and deaths related to the use of prescription opioids in the past few years [52, 53]. Four out of five patients obtaining Methodone Maintenance treatment in Toronto reported using non-medical prescription opioids at the time of admission [51].

The main limitation of this survey was the low response rate and unknown generalizability of the results. There may be multiple reasons for this low response rate, including lack of time among family physicians to complete the survey or the link to the survey in the News Brief being overlooked. However, it is also possible that the low response rate may be highlighting a more fundamental issue, a lack of interest among family physicians in the topic. Awareness campaigns and anti-stigma campaigns in addition to educational interventions may be required to affect the necessary change in the screening and treatment practices of substance use disorders by family physicians.

## Conclusions

The findings from the present study highlight the need for the integration of screening alcohol, tobacco and opioid use disorders using validated tools into the standard medical practice of family physicians. This is of particular importance for adolescents, for whom screening by family physicians is currently lower than adults. Furthermore, there is a need for training among family physicians to improve their knowledge on the currently available pharmacotherapies for substance dependences, particularly alcohol and opioid dependences. The findings from the current study suggest that there is a need for increased awareness on the importance of screening and treatment practices of substance use disorders among family physicians. Continuing efforts to establish new programs to increase physician awareness on the importance of screening of substance use disorders using validated tools and build physician knowledge on substance use disorder treatment approaches is greatly needed.
